# Investigating the Impact of Estrogen Levels on Voiding Characteristics, Bladder Structure, and Related Proteins in a Mouse Model of Menopause-Induced Lower Urinary Tract Symptoms

**DOI:** 10.3390/biom14091044

**Published:** 2024-08-23

**Authors:** Chenglong Zhang, Yuangui Chen, Lingxuan Yin, Guoxian Deng, Xiaowen Xia, Xiaoshuang Tang, Yifeng Zhang, Junan Yan

**Affiliations:** 1 Guangxi Key Laboratory of Special Biomedicine and Advanced Institute for Brain and Intelligence, School of Medicine, Guangxi University, Nanning 530004, China; 2028402006@st.gxu.edu.cn (C.Z.);; 2 Department of Urology, PLA Naval Medical Center, Naval Medical University, Shanghai 200052, China

**Keywords:** estrogen, voiding frequency, collagen deposition, *Piezo1*, *TRPV4*

## Abstract

Lower urinary tract symptoms (LUTS) are common in postmenopausal women. These symptoms are often linked to decreased estrogen levels following menopause. This study investigated the relationship between estrogen levels, alterations in bladder tissue structure, bladder function, and the incidence of urinary frequency. An age-appropriate bilateral ovariectomized mouse model (OVX) was developed to simulate conditions of estrogen deficiency. Mice were divided into three groups: a sham-operated control group, OVX, and an estradiol-treated group. The assessments included estrogen level measurement, urination frequency, cystometry, histological analysis, immunofluorescence staining, and real-time quantitative PCR. Additionally, we quantified the expression of the mechanosensitive channel proteins *Piezo1* and *TRPV4* in mouse bladder tissues. Lower estrogen levels were linked to increased voiding episodes and structural changes in mouse bladder tissues, notably a significant increase in *Collagen III* fiber deposition. There was a detectable negative relationship between estrogen levels and the expression of *Piezo1* and *TRPV4*, mechanosensitive proteins in mouse bladder tissues, which may influence voiding frequency and nocturia. Estrogen treatment could improve bladder function, decrease urination frequency, and reduce collagen deposition in the bladder tissues. This study explored the connection between estrogen levels and urinary frequency, potentially setting the stage for novel methods to address frequent urination symptoms in postmenopausal women.

## 1. Introduction

Lower urinary tract symptoms (LUTS), which include urinary frequency, urgency, nocturia, and overactive bladder, affect approximately 57–72.6% of women [1,2]. These symptoms are closely associated with estrogen deficiency, a critical factor in the progression of LUTS in aging women [3]. A thorough scientific investigation is necessary to explore the mechanisms that influence how varying levels of estrogen impact voiding frequency in this demographic. Recent studies by Christmas and Russo et al. demonstrated that estradiol can improve urodynamic parameters such as maximal bladder capacity, decrease non-voiding contractions of urethral muscles, and potentially alleviate symptoms like frequent urination [4,5]. Although some studies suggest a correlation between estrogen levels and urination frequency, the effectiveness of estrogen replacement therapy in addressing voiding frequency due to postmenopausal estrogen decline remains unclear [6]. This uncertainty emphasizes the importance of continued research into how estrogen levels affect the onset, progression, and potential relief of voiding frequency.

In addition to its role in urinary frequency symptoms, estrogen levels are connected to changes in bladder tissue structure. The lower urinary tract, including both urethral and bladder tissues, contains estrogen receptors [7,8]. Research has shown that estrogen deficiency can damage the bladder tissue structure, whereas estrogen supplementation was shown to increase bladder weight and vascular density within bladder tissues [9]. Moreover, the elasticity of urethral and bladder tissues, which affects various storage phase symptoms, was linked to estrogen levels [7]. Despite these insights, studies examining the link between estrogen levels and structural changes in bladder tissue are still limited.

The mechanosensitive channel *Piezo1* is widely distributed throughout the urinary system, primarily found in uroepithelial and mesenchymal cells, as well as in a subset of smooth muscle cells within bladder tissue [10]. As a mechanosensitive ion channel, *Piezo1* plays a vital role in detecting bladder stretch, which is crucial for urine storage and release [11]. The activation of *Piezo1* by bladder stretching triggers a sequence of events beginning with the flow of intracellular calcium ions, influencing sensations of bladder filling and the subsequent processes of urination [12]. Impaired *Piezo1* expression or activity can compromise bladder sensing mechanisms, leading to dysuria. Moreover, the involvement of *Piezo1* in urinary frequency is believed to arise from its role in regulating the response of bladder tissue to mechanical stimuli, impacting urine production and release [13,14]. Similarly, the transient receptor potential channel *TRPV4*, which responds to mechanical, thermal, and certain chemical stimuli, is found in bladder epithelial cells. It plays a role in monitoring bladder filling, thereby managing urine storage and release. The significance of *TRPV4* in maintaining normal bladder function is highlighted by the abnormal bladder activity seen in *TRPV4*-knockout mice [15,16,17,18]. The overactivation of *TRPV4* was associated with bladder overactivity and increased urinary frequency. Notably, *TRPV4* antagonists have shown effectiveness in alleviating stress-induced bladder issues by improving bladder capacity and reducing urinary frequency, suggesting that *TRPV4* is a potential therapeutic target for treating bladder disorders, including overactive bladder [19]. The link between menopausal LUTSs and the expression levels of these channel proteins warrants further investigation.

We hypothesize that the lower urinary tract of the OVX animals will deteriorate alongside estrogen deficiency and that changes in urinary frequency may correlate with collagen deposition and the expression of *Piezo1* and *TRPV4*. In this study, we established an induced surgical menopause model in mice, with and without bilateral ovary removal, to explore the relationship between estrogen levels and changes in urination frequency and bladder tissue structure.

## 2. Materials and Methods

### 2.1. Experimental Design of Animal Model

**Figure 1:**
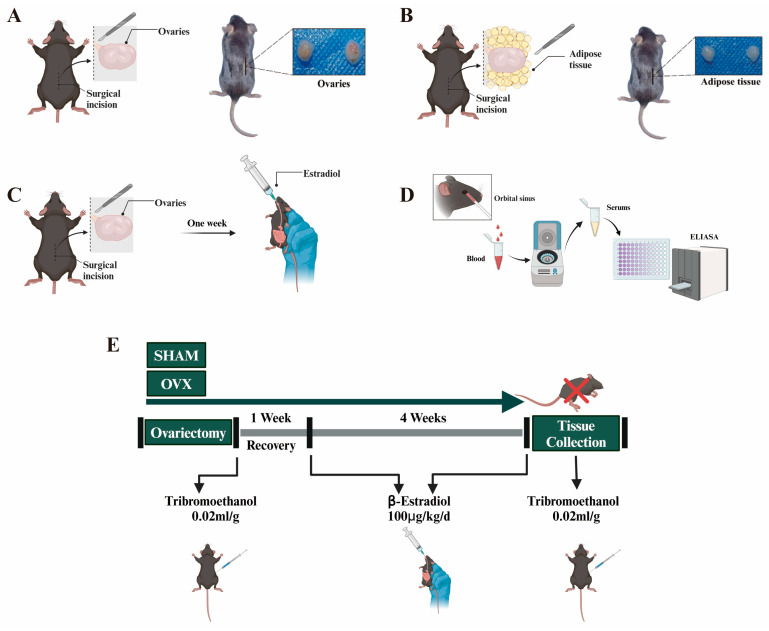
Development of mice experimental models: (**A**–**C**) implementation of experimental protocols in mice, involving bilateral oophorectomy (OVX), sham operation as a control (SHAM), and estrogen intervention; (**D**) procedures for blood sampling and estrogen level quantification using ELISA technique in mice; (**E**) details of the estrogen intervention protocol, including dosage and duration.

Twenty-four female *C57BL/6* mice, weighing between 17.0 and 18.0 g, were sourced from the animal center at GuangXi University. The experimental protocols received approval from the Committee for the Use of Experimental Animals at GuangXi University (approval number: Gxu-2021-1002). The mice were randomly allocated into SHAM, OVX, and E2 groups. For the OVX group, estrogen deficiency was induced through bilateral ovariectomy (OVX) surgery lasting 4 weeks. The procedure involved making a 1 cm incision on both sides of the spine of the female mice, followed by the removal of both ovaries. The OVX surgery was conducted under Tribromoethanol (M2910, Nanjing, China) anesthesia (Figure 1A). In the SHAM group, a similar incision was made, but only the adipose tissue surrounding the ovaries was removed (Figure 1B). The dosage of estrogen for the intervention was determined based on previous studies [20]. In the E2 group, β-estradiol (E8857, Sigma-Aldrich, Saint Louis, MO, USA) was administered at a dosage of 100 μg/kg/day via gavage starting one week after the bilateral OVX surgery (Figure 1C). Serum estrogen levels were measured using an ELISA four weeks post-OVX (Figure 1D). A urine study was conducted across all three groups within the 4-week period. Subsequently, the animals were euthanized, and their bladders were harvested for histological analysis. The experimental protocol adhered to the guidelines depicted in Figure 1E.

### 2.2. Evaluation of Estradiol Level

Serum was collected, and the serum estradiol concentration was determined using an estradiol ELISA kit (Elabscience, Wuhan, China) according to the manufacturer’s instructions [21]. The estradiol concentration was measured at 450 nm using an ELISA reader (Multiskan™ FC, Waltham, MA, USA).

### 2.3. Statistics on the Frequency of Urination

The frequency of urination was assessed starting 4 weeks post-OVX surgery. Mice were individually housed in 32 cm × 13.5 cm cages, and after a 48 h acclimation period, urine output was collected over 12 h light/12 h dark cycles using filter paper that covered the entire cage floor. To prevent damage to the filter paper, a plastic mesh with 0.8 cm diameter holes was placed over it. During the 48 h observation period, mice had access to water and food ad libitum. The frequency and intervals of urination were documented using video recording software. The urination events were carefully counted from the recorded videos. To ensure the reliability and accuracy of the data, the video analysis was consistently performed by the same individual. At the end of the 4-week period, the data underwent semi-quantitative analysis.

### 2.4. Bladder Cystometry (Urodynamics)

The cystometry was measured in accordance with the method previously described [19]. For cystometry, mice were anesthetized with tribromoethanol, which was administered intraperitoneally. Prior to surgery, abdominal hair was removed, and a 1 cm longitudinal incision was made in the lower abdomen to access the bladder. The skin and then the peritoneum were incised. After inspecting and moving aside surrounding fatty tissue, the bladder was exposed and carefully lifted using toothless forceps. A PE-10 catheter was inserted into the bladder wall via a syringe needle, secured with a knot, and routed subcutaneously to emerge at the neck area. The catheter’s end was sealed, and the abdominal incision was closed, layering skin, subcutaneous tissue, and muscle. Cystometry was performed two days post-catheterization using a pressure transducer (YPJ01, Chengdu, China) and flow pump connected to the catheter via a three-way tube. The cystometry assay involved infusing 0.9% saline at room temperature into the bladder at a rate of 0.03 mL/min. Bladder pressure was continuously recorded, analyzing at least five consecutive voiding cycles to assess intravesical pressure-related variables. Bladder capacity was calculated by multiplying the infusion rate by the duration until the onset of micturition, and maximum voiding pressure was defined as the peak pressure exerted by the detrusor muscle during voiding.

### 2.5. Masson Staining

The entire bladder weight was recorded. Frozen bladder sections, 5 μm thick, were air-dried and fixed in polyformaldehyde. Masson staining was performed using a commercially available Masson staining kit (Solarbio, Beijing, China), which stains collagen blue and smooth muscle red in each image [22]. Sirius Red staining was also conducted according to the Sirius Red staining kit protocol (Solarbio, Beijing, China), using a polarization microscope to distinguish different types of collagen within the samples [23]. Morphometric evaluation was performed on the samples. Using ImageJ/Fiji software version 1.53q (NIH, Bethesda, MD, USA), the total areas occupied by smooth muscle and collagen were quantified, and the ratio of collagen area to smooth muscle area was calculated.

### 2.6. Immunofluorescence Staining

The immunofluorescence staining procedure followed the methodology used in previous studies [11]. Whole bladder tissues were fixed in cold 4% paraformaldehyde in 0.1 M phosphate-buffered saline (PBS, pH 7.4) overnight. For antigen retrieval, tissue sections were boiled in sodium citrate buffer (pH 6.0) for 15 min. Subsequently, sections were permeabilized with 0.5% Triton X-100 at room temperature for 10 min, followed by three 5 min washes in PBS. The bladder sections were blocked in sheep serum working solution for 30 min. The slides were then incubated overnight at 4 °C with primary antibodies: rabbit polyclonal anti-E-cadherin (AF0131, Affinity, Changzhou, China) at 1:150, rabbit polyclonal anti-Piezo1 (DF12083, Affinity, Changzhou, China) at 1:150, and rabbit polyclonal anti-TRPV4 (DF8624, Affinity, Changzhou, China) at 1:150 in 0.5% BSA solution. After washing in PBS for 15 min, the slides were incubated with Goralite 488 goat anti-rabbit secondary antibodies (SA00013-2, Proteintech, Wuhan, China) diluted 1:400 at room temperature for 2 h. Finally, the slides were sealed with anti-fluorescence attenuation sealing tablets containing DAPI (Solarbio, Beijing, China). Images were captured using a fluorescent microscope (Olympus BX53F2, Tokyo, Japan). All images were taken under the same photography conditions to enable direct comparison between experimental animals and controls. To measure fluorescence signal intensity, the mean gray value was determined using ImageJ/Fiji software version 1.53q (NIH, Bethesda, MD, USA). The procedures were as follows: For a single-channel (monochromatic) fluorescence image, the gray value of each pixel indicated the fluorescence intensity at that point. The formula for calculating fluorescence intensity in a specific region was average fluorescence intensity (mean) = total fluorescence intensity of the region (IntDen)/area of the region (Area) (Mean: mean gray value; IntDen: integrated density).

### 2.7. Quantitative Real-Time PCR Assay

qRT-PCR was conducted following the manufacturer’s guidelines. Total RNA was extracted using a Trizol reagent (Invitrogen, Carlsbad, CA, USA). The reverse transcription was carried out with a high-capacity cDNA reverse transcription kit (Thermoscientific, Waltham, MA, USA). The cDNA was then amplified via PCR using SYBR-Green dye and the ABI Power SYBR-Green PCR Master Mix kit (Applied Biosystems, Foster City, CA, USA). Mouse primers were designed using Primer 3 software and synthesized by Sangon Biotech Co., Ltd. (Shanghai, China) (Table 1). The relative mRNA expression levels were determined using the 2^−∆∆Ct^ method, with mouse *β-actin* (Sangon, Shanghai, China) serving as the internal control. Each experiment was independently performed three times, with the averages taken for further analysis.

### 2.8. Statistical Analysis

Data analysis and graphing were performed using GraphPad Prism (Version 9.0, San Diego, CA, USA). Results are presented as means ± standard deviation (SD). Normality tests were conducted for all experimental datasets. Independent data were analyzed using one-way analysis of variance (ANOVA) with Tukey’s post hoc test for multiple comparisons if the data followed a normal distribution. If not, the non-parametric Kruskal–Wallis test with Dunn’s post hoc test was employed; *p* values < 0.05 were considered statistically significant: * < 0.05, ** < 0.01, *** < 0.001, **** < 0.0001.

## 3. Results

### 3.1. Establishment and Validation of Animal Models

Compared to the SHAM group, the mice in the OVX group showed a significant reduction in serum estrogen levels after four weeks of model establishment, as detailed in Table 2 (*p* < 0.0001). At the same point, estrogen levels in the E2 group matched those of the SHAM group, indicating that OVX effectively reduces estrogen levels. The administration of E2 notably increased circulating estrogen levels in mice. There was also a marked increase in body weight in the OVX group compared to the SHAM group (*p* < 0.001). Additionally, bladder weight in the E2 group was significantly higher than in the SHAM group (*p* < 0.01).

### 3.2. Urination Frequency in Mice Correlates with Estrogen Levels

**Figure 2:**
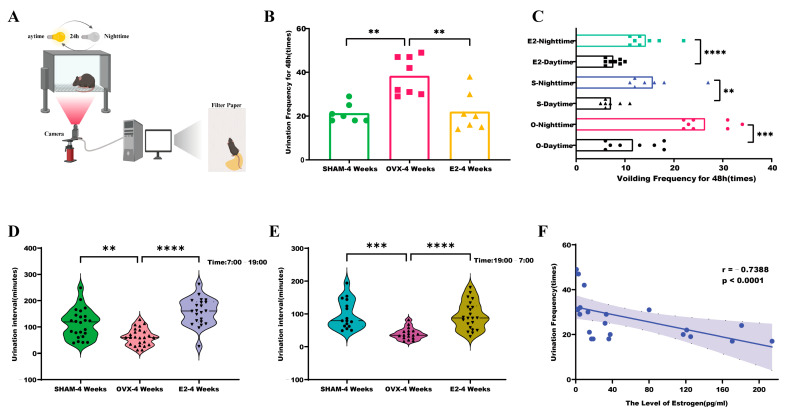
Association of estrogen fluctuations with altered urinary frequency in mice: (**A**) methodology for assessing urinary behavior in mice; (**B**) comparative analysis of voiding frequency over 48 h in OVX, SHAM, and E2 groups; (**C**) assessment of diurnal variations in 48 h urinary frequency among OVX, SHAM, and E2 groups after 4 weeks of model establishment. The number of data points representing the time intervals between adjacent micturition events across three mouse groups; (**D**,**E**) investigation of urination intervals (daytime and nighttime) in OVX, SHAM, and E2 groups; (**F**) correlation analysis between estrogen levels and voiding frequency. R, Pearson correlation coefficient; *p*-value, two-tailed *p*-value of the Pearson correlation. Statistical significance denoted as ** *p* < 0.01; *** *p* < 0.001; **** *p* < 0.0001.

Using the behavioral paradigm shown in (Figure 2A), the 48 h urination frequency was monitored and recorded for mice from the three groups four weeks post-establishment of the model. The OVX group displayed a statistically significant increase in 48 h urination frequency compared to the SHAM group (*p* < 0.01) (Figure 2B). It is important to note that the nocturnal/daytime phases correspond to awake/sleeping phases for mice. Notably, among the three groups, nocturnal urination frequency was highest in the OVX group and was significantly elevated compared to daytime urination frequency (*p* < 0.001) (Figure 2C). This increase in nocturnal urination frequency was reduced following estrogen treatment, as observed in the E2 group. Moreover, the intervals between urinations were significantly shorter for the OVX group during both daytime and nighttime compared to the SHAM group, a trend that was regressed with estrogen treatment (Figure 2D,E). Figure 2D,E display the number of data points representing the time intervals between adjacent micturition events across three mouse groups. The correlation between the level of estrogen and the urination frequency was analyzed using the Pearson correlation analysis. A negative correlation was found between the frequency of urination in mice and their estrogen levels (*p* < 0.0001, Figure 2F).

### 3.3. Bladder Function Is Correlated with Estrogen Levels in Mice

**Figure 3:**
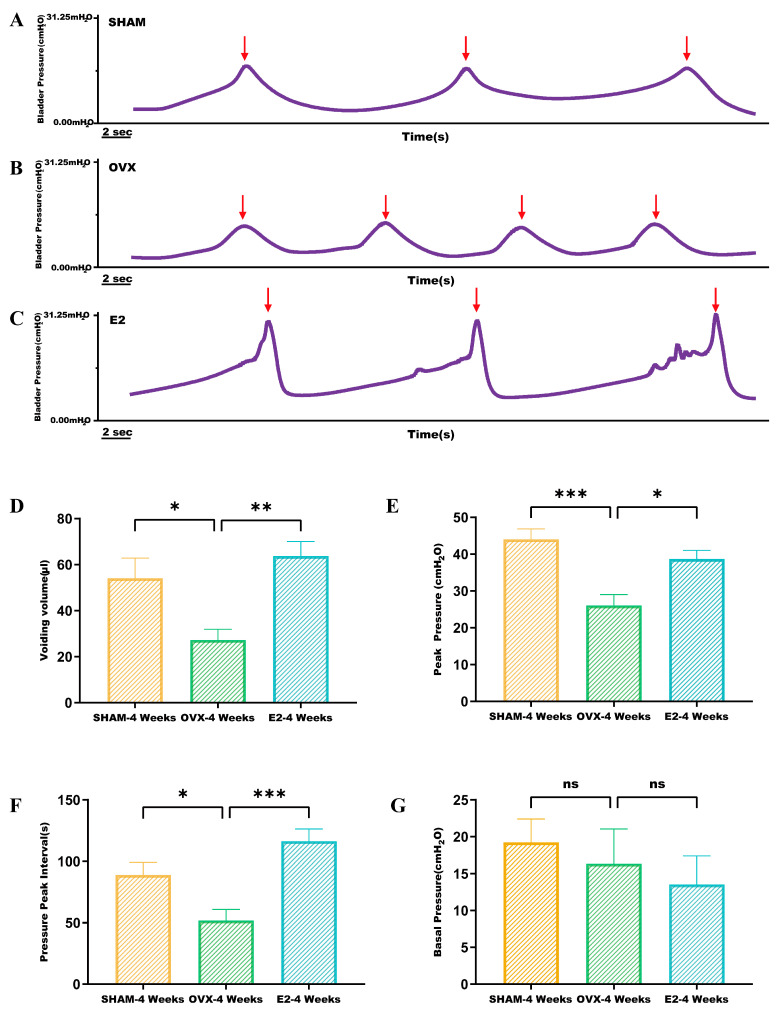
Urodynamic analysis was conducted to evaluate cystometric parameters in three groups of mice, namely SHAM, OVX, and E2: (**A**–**C**) cystometry in SHAM, OVX, and E2 group mice recorded changes in intravesical pressure, voiding time, and bladder contractions (arrows); (**D**–**G**) comparison of voiding volume, peak pressure, pressure peak interval, and baseline pressure of mice in SHAM, OVX, and E2 groups. Statistical significance denoted as * *p* < 0.05; ** *p* < 0.01; *** *p* < 0.001; ns indicates non-significant.

Bladder function assessments, based on urodynamic parameters such as peak pressure, voiding frequency, baseline pressure, and bladder capacity, are depicted in Figure 3. Mice in the SHAM group exhibited regular and steady voiding patterns, as shown in Figure 3A. In contrast, mice in the OVX group demonstrated an increased voiding frequency, which decreased following estrogen intervention (Figure 3B,C). Peak bladder pressure, indicated by arrows, was lower in the OVX group compared to the SHAM and E2 groups (Figure 3E). A comparison between the SHAM and E2 groups with the OVX group showed a significant reduction in bladder capacity in the OVX group (*p* < 0.05; *p* < 0.01) (Figure 3D). As depicted in Figure 3F, the interval between micturitions was shorter in the OVX group compared to the SHAM group (*p* < 0.05). However, after estrogen intervention, the interval between micturitions increased (*p* < 0.001). Baseline bladder pressure changes showed no significant correlation with estrogen levels across the groups (Figure 3G). Collectively, these findings suggest that bladder capacity and function are compromised in OVX mice but improve with estrogen intervention, indicating a relationship between estrogen levels and bladder function.

### 3.4. Estrogen Intervention Decreases Collagen Deposition in Bladder Tissue

**Figure 4:**
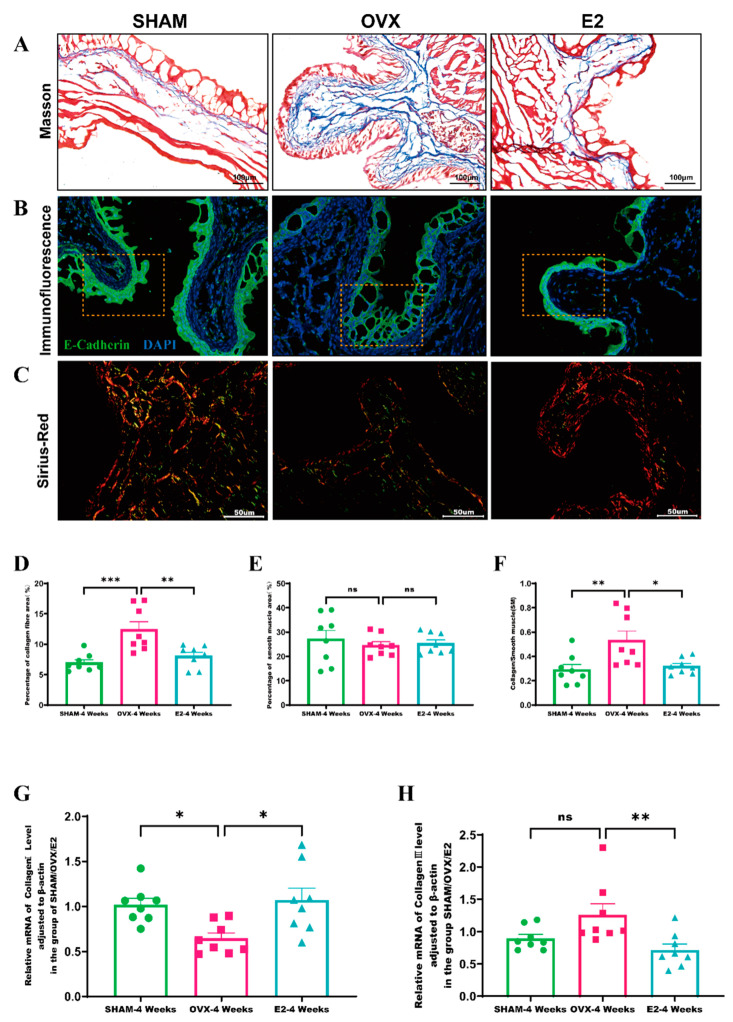
Alterations in bladder histology attributable to estrogen level variations: (**A**) Masson’s trichrome staining of bladder tissues from OVX, SHAM, and E2 group mice. This technique demarcates muscle fibers in red, collagen fibers in blue, and uroepithelial tissue in pink; (**B**) Immunofluorescence staining for E-cadherin in bladder tissues of mice in OVX, SHAM, and E2 groups (yellow dotted box); (**C**) Sirius Red staining applied to bladder tissues of mice in OVX, SHAM, and E2 groups for collagen detection; (**D**–**F**) comparative evaluation of the collagen fiber area, smooth muscle area, and the collagen-to-smooth muscle ratio in bladder tissues of each group; (**G**,**H**) quantitative analysis of *Collagen I* and *Collagen III* in bladder tissues across all groups. Statistical significance denoted as * *p* < 0.05; ** *p* < 0.01; *** *p* < 0.001; ns indicates non-significant.

Masson staining indicated that in the bladder tissue of mice from the SHAM group, smooth muscle fibers were neatly arranged, with scattered collagen fibers distributed sparsely between the uroepithelium and the smooth muscle layer. Conversely, in the OVX group, the arrangement of these fibers differed. The bladder tissue of mice in the OVX group exhibited numerous disorganized collagen fibers between the uroepithelium and the smooth muscle layer. However, in the E2 group, the collagen fibers in the bladder tissue significantly declined following estrogen intervention (Figure 4A).

**Figure 5:**
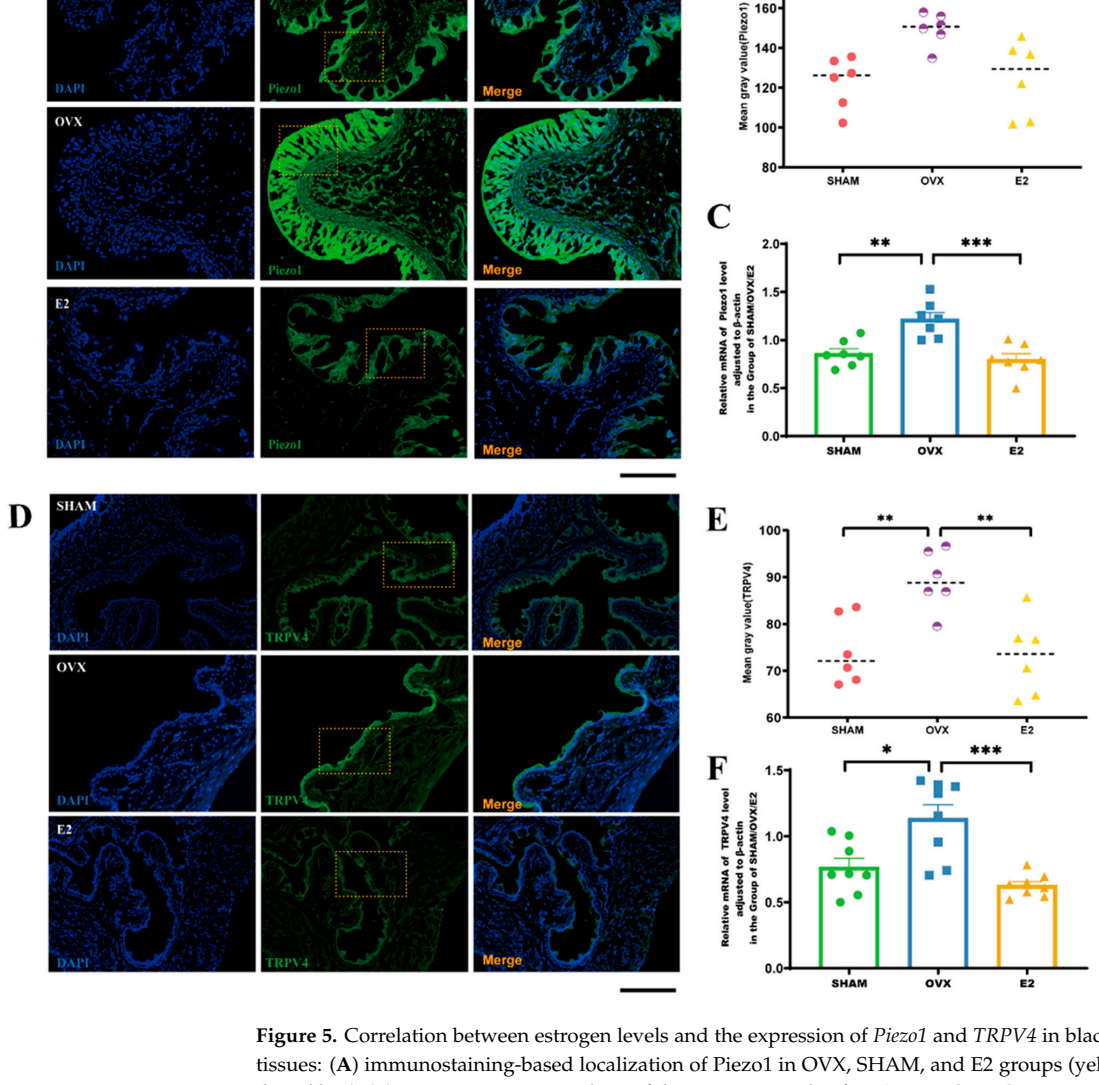
Correlation between estrogen levels and the expression of *Piezo1* and *TRPV4* in bladder tissues: (**A**) immunostaining-based localization of Piezo1 in OVX, SHAM, and E2 groups (yellow dotted box); (**B**) semi-quantitative analysis of the mean gray value for *Piezo1* in OVX, SHAM, and E2 groups; (**C**) assessment of relative mRNA levels of *Piezo1*, normalized to β-actin, in OVX, SHAM, and E2 groups; (**D**) immunostaining-based distribution of *TRPV4* in OVX, SHAM, and E2 groups (yellow dotted box); (**E**) semi-quantitative analysis of the mean gray value for *TRPV4* in OVX, SHAM, and E2 groups; (**F**) evaluation of relative mRNA levels of *TRPV4*, adjusted to β-actin, in OVX, SHAM, and E2 groups. Statistical significance indicated as * *p* < 0.05, ** *p* < 0.01, *** *p* < 0.001. Scale bar: 50 μm.

In the SHAM group mice’s bladder tissue, *E-cadherin* was predominantly located at the uroepithelial cell junctions. In contrast, the OVX group showed lower *E-cadherin* expression and weaker fluorescence intensity. Estrogen intervention restored *E-cadherin* fluorescence intensity in the uroepithelium to levels similar to those in the SHAM group (Figure 4B). Meanwhile, semi-quantitative analysis was performed to assess the proportion of collagen fibers and smooth muscle [24] tissue, as well as the ratio of collagen fibers to SM. Four weeks after model establishment, there was a significant increase in the percentage of collagen fibers in the bladder tissue of the OVX group compared to the SHAM group (*p* < 0.001). The percentage of collagen fibers significantly declined following estrogen intervention (*p* < 0.01) (Figure 4D). However, no significant changes were observed in SM composition within the bladder tissues of the three groups (Figure 4E). The ratio of collagen fibers to SM in the bladder tissue was significantly higher in the OVX group than in the SHAM group (*p* < 0.01), and this ratio decreased after estrogen intervention (*p* < 0.05) (Figure 4F). Sirius Red staining was used to visualize the distribution of *Collagen I* and *Collagen III* in the bladder tissues of the three different mouse groups (Figure 4C). Additionally, qRT-PCR was employed to measure the transcript levels of Collagen types I and III in these groups. As shown in Figure 4 and Figure 5, the levels of beta-actin transcripts were stably detected, and the Ct values were 17.04 ± 0.220,17.02 ± 0.294 and 17.01 ± 0.291, respectively.

Bladder tissues from the SHAM and E2 groups showed lower transcript levels of *Collagen I* compared to the OVX group, as indicated in Figure 4G. Conversely, transcript levels of *Collagen III* were higher in the OVX group, as shown in Figure 4H. These findings suggest that decreased estrogen levels may contribute to increased collagen deposition in bladder tissues, predominantly involving *Collagen III* fibers. Moreover, estrogen seemed to reduce collagen deposition in bladder tissues, potentially slowing the progression of bladder tissue fibrosis.

### 3.5. Estrogen Intervention Decreases Transcript Levels of Piezo1 and TRPV4 in Mice Bladder Tissue

Previous studies have demonstrated that estrogen levels influence the structural composition of mice bladder tissue [9]. Given the potential association between estrogen levels and urothelial channel, the expression and transcription levels of two mechanosensitive channels, *Piezo1* and *TRPV4*, were compared across the three groups in mice bladder tissue. The results demonstrated that bladder tissues from the OVX group showed higher fluorescence intensity and greater expression of the mechanosensitive channel *Piezo1* (Figure 5A,B) and *TRPV4* (Figure 5D,E) compared to the SHAM and E2 groups (*p* < 0.05; *p* < 0.01). Additionally, the OVX group exhibited increased transcriptional levels of *Piezo1* and *TRPV4* in bladder tissues relative to the SHAM group (*p* < 0.01; *p* < 0.05). Conversely, transcript levels of *Piezo1* and *TRPV4* decreased following estrogen treatment (*p* < 0.001; *p* < 0.001) (Figure 5C,F). These results suggest a significant reversed correlation between estrogen levels and the transcript levels of mechanosensitive channels such as *Piezo1* and *TRPV4*.

## 4. Discussion

This study developed a bilateral OVX mouse model to simulate reduced estrogen levels in mice, establishing it as a critical tool for investigating the relationship between estrogen levels and various physiological and pathological processes [25,26,27]. Notably, an increase in urination frequency was observed in the bilateral OVX group, demonstrating a negative correlation between estrogen levels and urination frequency. Previous studies, including those by Junya Yoshida et al. and F. Aura Kullmann et al., reported increased urination frequency in rats four weeks post-bilateral ovariectomy and increased nocturnal urination five to six weeks post-procedure, respectively [28,29]. Our findings also highlighted a diurnal variation, with decreased urination frequency during the day and increased frequency at night, a pattern that became more pronounced with lower estrogen levels. Diverging from previous research, our study extended the observation window from 24 to 48 h, allowing for a more comprehensive evaluation of the relationship between estrogen levels and urination frequency.

To address the increased urination frequency in bilateral OVX mice resulting from lowered estrogen levels, estrogenic interventions were implemented. Specifically, β-estradiol was administered orally at a dosage of 100 μg/kg daily for four weeks, starting one week after ovariectomy. Our data suggested that estrogen intervention effectively restored urination frequency to pre-bilateral OVX levels. This finding aligns with the results of Liang and Willmann et al., who showed that β-estradiol administration improved voiding frequency in bilateral ovariectomized rats [30]. Moreover, numerous randomized controlled clinical trials have confirmed the efficacy of estrogen in alleviating symptoms such as nocturia, urgency, and bladder capacity irregularities, highlighting its role in improving frequent urination among postmenopausal women [31,32,33]. This study reinforces the link between estrogen levels and urinary frequency, substantiating the effectiveness of estrogenic intervention in reducing increased urinary frequency in OVX mice. Although the OVX model can simulate a state of declining estrogen levels, aging itself can cause changes in lower urinary tract symptoms, bladder tissue structure, and function. Therefore, our future research will include the study of voiding behavior, bladder tissue structure, and function in aged mice.

Estrogen levels can not only influence the frequency of urination in mice but also affect the structural integrity of bladder tissue, particularly in terms of collagen deposition. Postmenopausal alterations in bladder structure were partially attributed to changes in estrogen levels [9]. In this study, we observed collagen deposition in the bladder tissue of OVX mice. This finding is consistent with the observations of Nicole et al., who noted collagen deposition in bladder tissues of the OVX model, linked to bladder instability [34]. Similarly, a previous study has investigated the collagen to SM tissue ratios in bilaterally ovariectomized and normal bladders, finding a significantly higher ratio in the OVX model [35]. These results align with our current findings, indicating that estrogen levels predominantly influence bladder tissue structure by altering collagen amounts. Additionally, previous studies have shown that abnormal bladder tissue increases connective tissue deposition, which in turn may improve the formation of bladder trabeculae and reduce bladder tissue compliance [36,37,38]. In this study, the collagen fibers between the uroepithelium and bladder smooth muscle in the bladder tissues of mice in the OVX group were significantly increased compared to the SHAM and E2 groups. It was observed that estrogen intervention resulted in increased bladder capacity and maximum bladder pressure. Estrogen may reduce collagen deposition in bladder tissues and bladder function in mice. However, further research is needed to confirm the relationship between collagen deposition in bladder tissue and bladder function. The detrusor muscles and their innervation are particularly susceptible to long-term deficiencies in ovarian hormones, resulting in impaired contractility of the bladder’s urethral muscles [39]. Notably, declining estrogen levels lead to various changes in the genitourinary system, including hypoxia, reduced blood flow to the bladder mucosa, and increased permeability, all contributing to decreased bladder compliance and potential urinary frequency [40,41,42,43,44]. Previous studies have established a link between the mechanical properties of the bladder wall and its elastic and connective tissue content, particularly in the force-generating muscles of the bladder. These properties are believed to depend on collagen distribution [45,46]. Collagen types I and III are crucial for tissue tensile strength [47]. Our study explored the relationship between these collagen types and estrogen levels, specifically examining how estrogen correlates with the collagen fiber content in bladder tissue. We observed that estrogen levels were associated with both *Collagen I* and *Collagen III* fibers in the bladder but exhibited distinct behaviors. Specifically, in the OVX group, characterized by low estrogen levels, *Collagen I* fiber content and transcript levels were reduced, while *Collagen III* fiber content and transcript levels were elevated. These findings suggest that the decrease in estrogen levels predominantly led to the deposition of *Collagen III* fibers in the bladder tissues of mice. Research on the impact of altered estrogen levels on the content of and *Collagen I* and *III* fibers in bladder tissue is limited. A study by Trabucco et al. observed a significant decrease in *Collagen I* content in the periurethral tissues of postmenopausal patients, highlighting potential impacts on connective tissue tensile strength [48].

The present study aimed to investigate the relationship between collagen deposition and estrogen levels in mouse bladder tissues. The results suggest that ovariectomy (OVX)-induced decreases in estrogen levels are followed by increases in collagen deposition in mouse bladder tissues. This pathological change can be improved after estrogen intervention. Estrogen plays a vital role in maintaining the elasticity of the bladder and periurethral tissues, potentially inducing the hypertrophy of the bladder detrusor muscle and increasing myosin levels in bladder tissue, thereby improving bladder contractility [49,50,51]. Estrogen intervention also improved bladder vascular density and distribution, which in turn improved contractility and bladder compliance. However, the specific mechanism by which estrogen inhibits bladder collagen formation remains unclear. Existing studies have suggested that estrogen may exert a cardioprotective effect in cardiac vessels by inhibiting fibroblast proliferation and collagen deposition, potentially mediated by the release of nitric oxide from endothelial cells [52]. The primary collagen types in bladder tissue are *collagen I* and *collagen III*. *Collagen I* is an essential component of the extracellular matrix in bladder tissue, providing a fundamental structural element. It is closely associated with the mechanical strength of the bladder wall and plays a role in maintaining the shape and function of the bladder wall. Its synthesis and accumulation in bladder smooth muscle are critical for maintaining normal bladder function [53]. Recent studies have shown that in conditions such as chronic cystitis, excessive accumulation of collagen I may lead to fibrosis of the bladder wall, which in turn affects bladder compliance and results in dysfunction in urine storage and voiding. *Collagen III* is a component of the extracellular matrix in the bladder and plays a crucial role in maintaining the structural stability of the bladder wall by forming a fibrous network. This network helps preserve the structural integrity of bladder tissue under mechanical stress, thereby preventing long-term damage to the bladder wall; In pathological states, such as bladder fibrosis, the expression of *collagen III* increases, typically resulting in sclerosis and a decline in the functional capacity of the bladder wall. In such cases, *collagen III* contributes to pathological remodeling of the tissue, potentially worsening urinary storage function [54,55,56]. Our study’s findings suggest that a reduction in estrogen levels is associated with an increase in *collagen III* deposits in bladder tissue. Therefore, it can be hypothesized that estrogen deficiency may affect the structural integrity of the bladder, potentially leading to the development of bladder fibrosis. This may impair the bladder’s ability to store urine, resulting in symptoms such as frequent urination. In conclusion, estrogen levels are linked to collagen deposition in mouse bladder tissue, particularly affecting the expression and transcript levels of *Collagen III*.

This study illustrated a link between estrogen levels and changes in bladder tissue structure. It further explored how estrogen levels influence the expression of mechanosensitive channels. Various channels are present in the urothelium of bladder tissue, including the transient receptor potential channel (*TRPV1*, *TRPV4*, etc.) and the mechanosensitive channel. The mRNA levels of *TRPV4* and *Piezo1* are higher than those of *TRPV1* in both bladder uroepithelial cells with sensory signaling and bladder lamina propria mesenchymal cells [57,58]. *Piezo1* and *TRPV4* in the uroepithelium are critical for sensing bladder fullness [59]. As shown in the graphical abstract presentation, previous research has revealed that *Piezo1* and *TRPV4* increase uroepithelial Ca^2+^ entry, subsequently increasing ATP release and stimulating bladder sensory nerve fiber afferents [12,60]. Recent studies have indicated that the functions of *Piezo1* and *TRPV4* are regulated by clock genes, which are active during waking hours and dormant during sleep, aligning with circadian rhythms [61]. Therefore, the increased expression and elevated transcript levels of *Piezo1* and *TRPV4* in bladder tissues, induced by decreased estrogen levels, may be linked to increased voiding frequency and nocturia. *Piezo1* and *TRPV4* are both expressed in bladder smooth muscle tissue and in the uroepithelial cells of mouse bladder tissue. *Piezo1* is expressed in various cell types within the bladder wall, including smooth muscle and mesenchymal cells. In a mouse model, *Piezo1* activation was shown to closely relate to the regulation of voiding frequency and bladder function. Intervention targeting *Piezo1* can alter bladder responsiveness and, consequently, influence voiding behaviour [13]. In addition to its role in sensing bladder filling, *TRPV4* was demonstrated to directly affect the contraction of bladder smooth muscle. By regulating the muscle’s mechanical sensitivity, *TRPV4* plays a dual role in bladder storage and voiding. Its activation was shown to increase smooth muscle contractility by triggering the Ca²⁺ signalling pathway, thereby facilitating efficient bladder emptying [60]. Consequently, we included these two channel proteins in our study. In conjunction with our findings, suggesting that reduced estrogen levels could impair urinary epithelial barrier function, the expression and transcript levels of *Piezo1* and *TRPV4* channels in bladder tissue were evaluated. This assessment revealed a strong negative correlation between estrogen levels and the expression and transcript levels of *Piezo1* and *TRPV4*. Further research is needed to understand the impact of reduced estrogen on *Piezo1* and *TRPV4* expression and its association with urinary frequency and nocturia.

## 5. Conclusions

Our findings revealed a significant negative correlation between decreased estrogen levels and urinary frequency in mice. Decreased estrogen levels are associated with *Collagen III* deposition in mouse bladder tissue. Estrogen supplementation improved bladder function and tissue structure and reduced collagen deposition. Given that the *Piezo1* and *TRPV4* levels in bladder tissue increased significantly after OVX, these proteins may be associated with frequent urination and nocturia caused by estrogen deficiency.

## Figures and Tables

**Table 1:** Primers used for quantitative PCR.

Gene	Forward Primer (5′→3′)	Forward Primer (5′→3′)
*Piezo1*	ATCGCCATCATCTGGTTCCC	AGCTCCAAGGTGTGCTTCTC
*TRPV4*	TGAGCAGGCCGAGAAGTACA	AGTCCATCTAGGTCCGCAGT
*Collagen I*	ACGTAAGCACTGGTGGACAG	CAGGAGGGCCATAGCTGAAC
*Collagen III*	GAGGAATGGGTGGCTATCCG	TTGCGTCCATCAAAGCCTCT
*β-actin*	GCAGGAGTACGATGAGTCCG	AGTCCATCTAGGTCCGCAGT

**Table 2:** Physical indicators for the different experimental groups.

Variable	SHAM	OVX	E2
Serum estradiol level (pg/mL)	35.20 ± 17.21	1.80 ± 1.32 ****	38.18 ± 22.00
Water intake (mL/48 h)	9.50 ± 1.38	10.69 ± 3.42	8.00 ± 1.26
Body weight (g)	17.59 ± 0.58	20.22 ± 1.17 ***	19.13 ± 1.99
Bladder weight (mg)	17.00 ± 1.60	18.00 ± 2.60	25.00 ± 2.20 **
The ratio of bladder weight (mg)/body weight (g)	0.89 ± 0.13	0.90 ± 0.15	1.1 ± 0.14

OVX, surgical ovariectomy; E2, estrogen treatment; values are means ± SD. ** *p* < 0.01; *** *p* < 0.001; **** *p* < 0.0001 versus the SHAM group.

## Data Availability

Data are available upon request.
